# Preliminary study into the components of the fear-avoidance model of LBP: change after an initial chiropractic visit and influence on outcome

**DOI:** 10.1186/1746-1340-18-21

**Published:** 2010-07-30

**Authors:** Jonathan R Field, Dave Newell, Peter W McCarthy

**Affiliations:** 1 Private practice. Back2Health, 2 Charles Street, Petersfield, Hants, GU32 3EH, UK; 2 Anglo European College of Chiropractic, 13-15 Parkwood Road, Bournemouth. BH5 2DF, UK; 3 Welsh Institute of Chiropractic, University of Glamorgan, Pontypridd, CF37 1DL, UK

## Abstract

**Background:**

In the last decade the sub grouping of low back pain (LBP) patients according to their likely response to treatment has been identified as a research priority. As with other patient groups, researchers have found few if any factors from the case history or physical examination that are helpful in predicting the outcome of chiropractic care. However, in the wider LBP population psychosocial factors have been identified that are significantly prognostic. This study investigated changes in the components of the LBP fear-avoidance beliefs model in patients pre- and post- their initial visit with a chiropractor to determine if there was a relationship with outcomes at 1 month.

**Methods:**

Seventy one new patients with lower back pain as their primary complaint presenting for chiropractic care to one of five clinics (nine chiropractors) completed questionnaires before their initial visit (pre-visit) and again just before their second appointment (post-visit). One month after the initial consultation, patient global impression of change (PGIC) scores were collected. Pre visit and post visit psychological domain scores were analysed for any association with outcomes at 1 month.

**Results:**

Group mean scores for Fear Avoidance Beliefs (FAB), catastrophisation and self-efficacy were all improved significantly within a few days of a patient's initial chiropractic consultation. Pre-visit catastrophisation as well as post-visit scores for catastrophisation, back beliefs (inevitability) and self-efficacy were weakly correlated with patient's global impression of change (PGIC) at 1 month. However when the four assessed psychological variables were dichotomised about pre-visit group medians those individuals with 2 or more high variables post-visit had a substantially increased risk (OR 36.4 (95% CI 6.2-213.0) of poor recovery at 1 month. Seven percent of patients with 1 or fewer adverse psychological variables described poor benefit compared to 73% of those with 2 or more.

**Conclusions:**

The results presented suggest that catastrophisation, FAB and low self-efficacy could be potential barriers to early improvement during chiropractic care. In most patients presenting with higher psychological scores these were reduced within a few days of an initial chiropractic visit. Those patients who exhibited higher adverse psychology post-initial visit appear to have an increased risk of poor outcome at 1 month.

## Background

Trials comparing physical therapies, including chiropractic, to other types of care have generally found them to provide superior benefits for lower back pain patients, but often only marginally [1,2]. It has been suggested that this may, in part, be due to the presence of subgroups of patients that together fulfil the inclusion criteria of the study but react differently to treatment [3,4]. Given this possibility, if it were feasible to identify those patients presenting for treatment who are likely to fail to improve with 'standard' care then alternative management could be offered. It would also enable through further clinical trials the potential of constructing guidance for practitioners as to the best direction that this alternative management may take [3]. The importance of this line of enquiry is highlighted by the Cochrane Collaboration who have referred to the ability to group back pain according to likely response to treatment as the 'Holy Grail' of back research [5].

In a series of prospective trials looking for predictors of outcome in chiropractic patients the 'Nordic Back Pain Sub-population Program' examined 70 potential baseline factors. Five were found to negatively influence prognosis; total duration of LBP in the preceding year (> 30 days), gender (being female), leg pain, concomitant painful musculoskeletal complaints and receipt of social benefit [6-10]. Studies in the United Kingdom also found that duration of the presenting complaint and to a lesser extent being female significantly influenced outcome [4]. As with the Nordic studies nothing from the physical examination was found to be associated with differential outcomes, therefore suggesting that these factors may be unimportant in predicting outcome during a course of chiropractic management.

Studies using the general back pain population have similarly identified few physical factors capable of explaining why back pain in some individuals settles quickly whilst in others develops into more chronic conditions, often despite treatment. Psychological and social influences however have been found to have significant impact on response to treatment and the development of chronicity. A range of cognitive and affective domains have been linked to enduring back pain including beliefs that back pain is inevitably negative, depression, anxiety, catastrophisation (hopelessness, magnification and rumination regarding pain) and fear-avoidance beliefs [11-14]. This had led to a call for these factors to be taken into account alongside examination findings when deciding on the management plan for all LBP patients [15,16]. Early work that viewed LBP patients with higher psychosocial factors as more likely to fail with physical treatments has had success in identifying a subgroup not responding to physiotherapy, and also had some success when directing these to psychologically based treatments [17-19].

Despite the significant predictive value of psychosocial factors found in other patient groups, investigation of these factors in chiropractic patients indicate they are of less importance [20,21]. It has been suggested that this may be because patients choosing to present to a chiropractor generally have lower levels of potentially adverse psychological functioning [20].

Whilst few pre-treatment measures have yet been found which influences outcome, Axen et al., [7] have indicated that for patients presenting to chiropractors with either acute or persistent lower back pain, response to the first session of care is highly predictive. Those not gaining any change after one session were significantly less likely to report worthwhile benefit at follow up.

**Figure 1:**
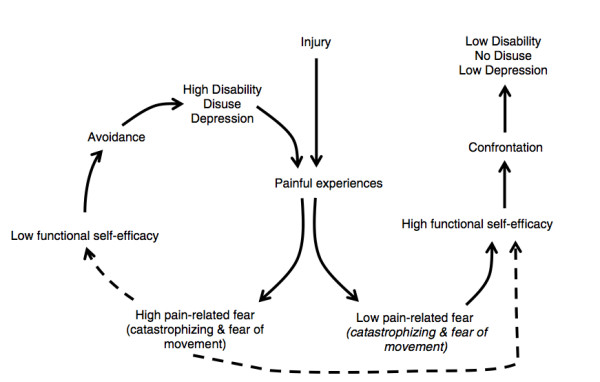
**Reprinted from European Journal of Pain, 11, Woby, Urmston, Watson, Self-efficacy mediates the relation between pain-related fear and outcome in chronic low back pain patients, 711-718, Copyright (2007), with permission from Elsevier **[46].

Several models exist to explain the influence of non-physical factors on the development of chronicity and treatment resistance. Amongst the more widely investigated is the fear-avoidance beliefs model introduced by Lethem et al [22,23] and developed specifically to relate to LBP by Vlaeyen et al. [24]. It has considerable support in the literature and has become the basis for treatment protocols drawing on a cognitive-behavioural approach [25,26]. This model suggested that an individual's behavioural response to LBP falls between the extremes of getting on with all daily activities despite the pain (confronting it), or avoiding all tasks that may (in their mind) cause further pain or (re)injury. However, there have been criticisms regarding the quality of evidence, and the underpinning relationship between altered behaviour and disability has been called into doubt by Pincus et al [27]. The restriction of activity by the 'avoiders' is purported to predispose them towards reducing fitness (disuse), depression, persisting pain and increasing disability (Figure 1). The Fear avoidance model as relates to LBP is made up of a number of components including: back beliefs, catastrophising, fear avoidance beliefs and self-efficacy

Some back pain patients hold the belief that there is something inevitably negative about back pain, and in a secondary analysis of the data from the BEAM UK [28] study (comparing manipulation, exercise and GP care) Underwood et al [29] reported that patients who held negative back beliefs tended to have a poorer prognosis than those who did not.

Catastrophising is considered to be an exaggerated and negative orientation toward pain stimuli and pain experience; individuals who catastrophise expect that they will cause a new episode of pain or injury, thus fuelling fear of motion [30,31]. Catastrophisation in back pain patients has been seen to be both a significant and independent predictor of response to treatment and development of chronicity [31-33]. Within the fear-avoidance model, catastrophising is postulated to affect an individual by increasing fear of activity and possibly increasing the risk of subsequent psychological distress and depression.

The term fear-avoidance belief (FAB) refers to aberrant or excessive concerns individuals may hold regarding the likelihood of their causing (re)injury by performing activities. These beliefs are significant when they cause people to change their activities (fear-avoidance behaviour). FAB's are seen to be predictive of outcomes of care where these are measured by disability, but not by severity of pain, [34-39]. Lower back patients with high FAB's have a poorer response to physical treatments than those with less [28,35,40,41]. Fear of movement may encourage LBP patients to tend towards avoidance of activity and thus enter the cycle postulated in the fear-avoidance model.

Self-efficacy is the belief in one's capabilities to organise and execute the actions required to manage prospective situations [42]. It has been suggested that for people who feel that they can accomplish tasks, where this belief is stronger than any FAB they hold, they will confront their pain, and more than likely, remain active. This could make them less likely to become locked into the cycle of fear, avoidance, disuse and pain [43,44]. Trials investigating this possibility appear to confirm that high self-efficacy is protective for individuals suffering LBP, and may moderate the impact fear beliefs have in inducing fear-avoidance behaviour [45,46]. This has led Woby et al. [46] to suggest self-efficacy as an important addition to the Fear-Avoidance model.

Although considerable evidence exists that supports the impact of psychological variables on recovery in LBP patients generally, limited investigation of these domains in chiropractic patients suggests they may be of less importance [20,21]. This has been suggested to be due to the observation that patients choosing to present to a chiropractor generally have lower levels of potentially adverse psychological functioning [20]. However, although the mean level of adverse psychology may well be lower than the wider LBP population it remains possible that of those chiropractic patients that present with significant psychological distress this may still present a barrier to recovery.

The aim of this study therefore, was to further investigate the components of the fear-avoidance beliefs model within a chiropractic LBP patient population. In particular we aimed to ascertain to what extent fear-avoidance belief components pre and post an initial visit are associated with outcome at 1 month follow up.

## Methodology

### Patient recruitment

During 2009, consenting new patients with lower back pain as their primary complaint presenting for chiropractic care to one of five clinics (nine chiropractors) were asked to complete questionnaires before their initial visit (pre-visit) and again just before their second appointment (post-visit).

Follow up patient global impression of change (PGIC) scores were ascertained 1 month after the initial consultation concerning patients perceived improvement

All patients fulfilled inclusion criteria, which consisted of presenting as a new patient to a chiropractor with lower back pain as the main complaint (with or without leg symptoms), and being accepted for care.

### Pre and Post initial visit measures

#### Fear-avoidance Beliefs Questionnaire (FABQ)

The Fear-avoidance beliefs questionnaire developed by Waddell et al. [47] has been widely used by researchers to assess the beliefs patients hold regarding the significance of pain they may feel when performing activities. It has two sub-scales one for general physical activity and the other for work related tasks. Test-retest reliability and internal consistency (Cronbach's α = 0.75, test retest ICC = 0.72 0.90) have been confirmed in previous studies [48-51].

The work sub-scale has been shown to relate predominately to work related outcome measures. Because our outcome determinant did not directly include return to work, as an earlier study has found few patients from samples similar to ours taking sickness absence, and to reduce the overall size of our test instrument, we chose to use just the physical activity sub-scale [20].

As further support for use of this sub scale it was shown to predict low back disability in patients attending an orthopaedic outpatient clinic (adjusted R^2 ^0.46, p < 0.001) [47]. As used here, it consisted of five items, with one being discarded for scoring, each having a Likert scale anchored via 'completely disagree' and 'completely agree' (0 and 6 respectively) giving a total score range of 0-24. Higher scores indicate more fear avoidance beliefs.

#### Catastrophising sub scale of the Coping Strategies Questionnaire (CSQ)

The catastrophising sub-scale of the Coping Strategies Questionnaire developed by Rosenstiel and Keefe [52] asks patients to rate the frequency of catastrophic thoughts they have regarding their pain. It has been shown to have high test-retest reliability and good internal consistency (Cronbach's α = 0.78 0.91, test retest = 0.81) [52,53].

The CSQ consists of six items with a score range of 0-36 with higher scores indicating more catastrophic thinking. It is scored on a seven point Likert scale with zero being anchored by 'Never' and 6 as 'Always'.

#### Back Belief Questionnaire (BBQ)

The Back Belief Questionnaire was developed to assess the beliefs a patient may hold about back pain, particularly that it is an inevitably negative process. It has reliable psychometric properties (Cronbach's α = 0.7, intra-class correlation coefficient = 0.87) [54].

The BBQ consists of nine items with a score range from 9-45. It uses a Likert scale anchored at 1 by 'completely agree' and 5 by 'completely disagree' to rate statements such as 'Back trouble must be rested' and 'Once you have back trouble there is always a weakness'. Lower scores indicate more negative beliefs regarding back pain.

#### Functional Self-efficacy (PSS)

The functional sub-scale of the Pain Self-efficacy Scale was developed by Anderson et al. [55]. It enquires how confident patients feel about their ability to complete tasks or participate in activities such as 'Walk half a mile on flat ground' and 'Engage in social activities'.

Having adapted it to a nine point Likert scale with 0 anchored to 'very uncertain' and 8, 'very certain', Woby et al [46] found it had excellent internal consistency (Cronbach's α 0.88) and good test-retest reliability (intra-class correlation coefficient = 0.88 [CI; 0.80-0.93]). In its adapted form it consists of nine items with a score range of 0-72 with higher scores indicating higher belief in ability to complete tasks.

#### Pain intensity

An eleven point numerical rating scale (NRS) with 0 = No pain and 10 = Worst pain possible was used to assess patients perceived pain intensity. It has been described as having a Cronbach's α of 0.82 and intra-class correlation coefficient > 0.8 [56]. In a chiropractic setting it has been show to be at least as responsive as other pain measures [57].

#### Outcome measure

The Patients Global Impression of Change (PGIC) is widely used as an outcome measure and has been described within a chiropractic patient population [58]. This scale consists of 7 categories; (1) No change (or condition has become worse), (2) Almost the same, hardly any change at all, (3) A little better, but no noticeable change, (4) Somewhat better, but the change has not made any real difference, (5) Moderately better and a slight but noticeable difference, (6) Better and a definite improvement that has made a real and worthwhile difference, (7) A great deal better, a considerable improvement that has made all the difference.

This has advantages over other outcome tools in asking about the impact of any improvement within the context of individual patient's lives. It therefore measures outcomes in terms of what individual patients feel is important. Despite concerns as to patients being biased as to their current status and recall of initial status these outcomes have been widely used and recommended for their relevance to meaningful change for the patient [59,60].

### Data Analysis

All data was tested for parametric distribution using a Kolmogrov-Smirnov test. For data not parametrically distributed (CSQ scores) non-parametric tests were used including correlation analysis. Logistic regression was used to calculate both univariate and adjusted odds ratios. The results were analysed using SPSS v18.0.

## Results

Seventy-five patients were recruited to the study, and completed the baseline questionnaire. Of these, three were incomplete and one patient was found not to have back pain as their main complaint, resulting in a sample size of 71. The sample had a mean age of 42.3 (SD 14.4) years with a range 19 to 82, with 46.5% (n = 33) being male and 39.4% (n = 28) having had their pain for over 1 month. There was an average interval of 4.3 days (SD 2.7) between the first and second appointments with a range of 1 - 12 days. Four patients did not complete post visit questionnaires, two because they did not attend their next booked appointment and two for administrative reasons, resulting in 65 completed post visit questionnaires. Forty-eight correctly completed PGIC questionnaires at 1 month, which translated to a 67.6% follow up.

Table 1 shows the results for pre and post initial visit scores. It is clear that improvement occurred in the context of these domains with catastrophising, fear avoidance and pain scores significantly reducing, and self-efficacy increasing. However, back belief scores did not change significantly.

**Table 1:** Pre and post initial visit mean scores

Variable	Pre Visit		Post Visit		p value (pre-post)
	**Range**	**Mean (SD)**	**Range**	**Mean (SD)**	

PSS	0-72	50.8(18.0)	0-72	52.9(19.3)	0.038*
CSQ	0-36	7.9(8.1)	0-24	5.5(6.9)	0.001**
FABQ	1-23	14.6(5.5)	0-23	11.1(5.2)	0.001*
BBQ	15-24	30.4(7.5)	14-45	31.0(7.7)	ns*
Pain (NRS)	1-10	6.1(2.2)	0-9	4.2(2.2)	0.001*

PSS = Pain related self-efficacy, CSQ = Catastrophising, FABQ-Fear Avoidance, BBQ = Negative back beliefs, * = Paired Sample T Test, ** = Wilcoxon's Signed Ranks Test, ns = not significant

Pre and post scores were investigated for any association with outcome (PGIC) at 1 month. The results for this analysis are presented in Table 2. For pre visit scores it can be seen that only catastrophic thinking (CSQ) was significantly associated with outcome. In contrast at post-visit both self-efficacy (PSS) and back beliefs (BBQ) in addition to CSQ scores, significantly correlated with outcome, albeit rather weakly.

**Table 2:** Correlation of pre and post visit scores to PGIC outcome

Variable	Correlation Coefficient (Spearman's rho)#	
	Pre Visit	Post Visit
PSS	0.03	0.33*
CSQ	-0.35*	-0.47**
FABQ	-0.03	-0.16
BBQ	0.17	0.34*

* = p < 0.05, ** = p < 0.01; PSS = Pain related self-efficacy, CSQ = Catastrophising, FABQ-Fear Avoidance, BBQ = Negative back beliefs; PSS and PGIC (high score is desirable); CSQ, FABQ and BBQ (low score is desirable)

In order to ascertain any predictive utility of post visit psychological scores, logistic regression analysis were performed with dichotomised PGIC as the dependant variable (scores > 5 on the PGIC were taken as improvement). The first analysis included all raw post-visit psychological scores in the model. This first model significantly predicting 1 month outcome (omnibus chi-square = 18.9, df = 2, p < 0.001). This model accounted for between 33% and 47% of the variance in PGIC with a sensitivity of 91.4% and a specificity of 53.8%. Regression coefficients reveal that a decrease of one point on CSQ (decreased adverse psychology) is associated with a decrease in the odds of poor outcome (OR 0.85 (95% CI 0.73 0.94). In addition, a 1 point reduction in PSS score (increased adverse psychology) was associated with an increased risk of poor outcome, although only marginally (OR-1.05 (95% CI 1.00-1.09)).

In a second and otherwise identical analysis, scores for PSS, CSQ, FABQ and BBQ were dichotomized about pre-visit group medians. Initially a univariate analysis revealed no significantly increased odds of poor outcome for high pre-visit scores for any variable. However, post-visit high scores were each associated with a raised risk of poor outcome (Table 3). Subsequently a forward LR binomial analysis including all post visit variables was carried out and also significantly predicted 1 month outcome (omnibus chi-square = 22.5, df = 2, p < 0.001). In this adjusted model, only CSQ and FAB remained as significant prognostic predictors. The model accounted for between 37% and 53% of the variance in PGIC with a sensitivity of 71% and specificity of 89%. In this model higher post visit CSQ and FAB scores were associated with poor outcome at 1 month (OR of 13.5 (95% CI 2.5-71.4), OR 8.7 (95% CI 1.4-55.0) respectively).

**Table 3:** Crude Odds Ratios for post visit scores associated with poor outcome at 1 month

Variable (category)	Odds Ratio	95% CI
PSS (low)	3.8	1.1 to 14.2
CSQ (high)	19.2	4.2 to 100.0
FABQ (high)	14.3	2.9 to 76.0
BBQ (high)	5.7	1.1 to 29.1

PSS = Pain related self-efficacy, CSQ = Catastrophising, FABQ-Fear Avoidance, BBQ = Negative back beliefs

In light of the above results, an assessment was made to ascertain the proportion of improved and not improved patients with 0, 1, 2 3 or 4 psychological variables (PSS, CSQ, FABQ, BBQ) that were raised above the pre-visit group median when assessed post-visit (Table 4).

**Table 4:** Effect of the number of high psychological scores post visit on the proportion of patients improving at 1 month

Number of high variables	Improved	not Improved (%)
0	15	0 (0%)
1	13	2 (13%)
2	2	5 (71%)
3	2	5 (71%)
4	1	3 (76%)

It is apparent from table 4 that individuals possessing more than one adverse psychological variable post-visit did poorly at 1 month compared to those with one or less. Of the 30 patients with one or less raised psychological variables post visit only 2 (7%) felt they had not improved significantly at 1 month. This contrasts with 13 (72%) of the 18 with 2 or more raised variables. This translates to an increased odds ratio of 36.4 (95%CI 6.2-213.0) for having a poor 1 month prognosis in those with 2 or more higher post visit psychological variables. The width of the confidence intervals is likely to be a consequence of the limited sample size

In this study, adverse psychological indices in patients post the initial visit seems important to subsequent outcomes. However, in the group of practices from which the trial patients were recruited it is unusual for new patients to receive no hands-on care at their first session with a chiropractor. Indeed a review of the case files indicated that all but four of the patients in this study received treatment during their first visit and it is plausible therefore that treatment during this visit may have significantly reduced pain and that it is reduction in this parameter that successfully modifies psychological factors pre to post visit. In order to investigate the impact of early improvement in pain on changes pre to post visit correlation between change in pain and change in psychological scores were calculated. (Table 5). The result of this analysis suggests that no significant correlation exits between change in pain and changes in either catastrophising or fear-avoidance beliefs. This supports the view that improvements in catastrophic thinking and fear-avoidance beliefs in the those patients recruited for this study were unlikely to be solely mediated by a change in pain. In contrast, there does appear to be a relationship between a decrease in pain perception and a rise in self-efficacy (PSS).

**Table 5:** Correlation coefficients between psychological and pain change scores

Comparison	Correlation Coefficient*	p value (2-tailed)
Δ PSS v Δ Pain	0.346	0.006
Δ CSQ v Δ Pain	0.241	0.062
Δ FABQ v Δ Pain	-0.067	0.614

* Pearsons, PSS = Pain related self-efficacy, CSQ = Catastrophising, FABQ-Fear Avoidance, BBQ = Negative back beliefs

## Discussion

The results from this trial largely confirm previous studies involving chiropractic patients in finding that the assessment of a patients psychological profile before an initial consultation is not helpful in identifying those less likely to improve [20,21]. The measurement of catastrophisation was an exception however, being moderately correlated with patient reports of improvement. This is the first published study describing the effect of catastrophisation in a chiropractic population. In this preliminary and limited study the majority of patients presenting at baseline with higher PSS, CSQ or FABQ scores displayed beneficial changes between baseline and follow up. Importantly, those who have 2 or more high psychological scores post-visit were more likely to have a poor prognosis. Despite the wide confidence intervals, probably as a result of the small sample size, the results presented here suggest that the persistence of higher psychological scores, beyond the immediate initial consultation may provide a significant barrier to improvement during chiropractic care.

That assessment of psychological variables after a consultation is more predictive of outcome is a potentially important observation. The literature to date in this population indicates that few if any modifiable prognostic factors are identifiable at baseline [20,21]. One reason for this may be that potential barriers to recovery do not emerge until attempts to ameliorate them have been applied. In other words, although patients may have higher baseline scores across a range of potential predictors it is the resistance to early change of these parameters, not the baseline scores themselves that could be potentially prognostic. Indeed Axen et al [7] have shown that changes at the 1^st ^visit can be significantly predictive of outcome. It is possible that psychological factors are useful components that when used alongside others can mark early change and therefore indicate greater capacity for recovery in sub-groups of LBP patients. Treatment packages currently suggested for those at higher risk of persisting LBP are typically resource intensive [61]. However, if sub grouping for care pathway purposes was conducted after an initial consultation then only those at continued higher risk would be considered, potentially enabling a more appropriate targeting of resources.

The relationship between changes in pain and improving self-efficacy was in contrast to other psychological metrics measured. Self-efficacy towards an activity is an appraisal of actual physical ability, the additional pain anticipated in performing the task and the individual's belief in their ability to tolerate this extra pain. Therefore with lower overall pain being related to lower anticipated pain for any specific task, it is not unexpected for reduced pain to be related to an increase in self-efficacy [44]. On the other hand, an absence of any relationship between changes in pain and change in FAB scores is in concordance with a strong body of work indicating that there is only a limited relationship between pain and fear-avoidance beliefs [34-36,39,62-64]. In contrast however, one might have expected a relationship between pain and catastrophising as in both patient and non-patient groups, catastrophising has been shown to be related to pain. For example a dose dependant pattern has been reported whereby an increase in catastrophisation is mirrored by a rise in reported pain [31,65]. It unclear why this effect is not seen in the presented study and it is possible that the few days between initial visit and post visit assessment were not enough for this relationship to become manifest. Further study investigating the time dependence of this effect may clarify this issue.

In this study, given the lack of relationship between changes in catastrophising and FAB versus pain, it maybe suggested that something other than physical treatment may account for some of the improvement seen. It is possible that providing time for patients to talk about their problem and for them to be examined by someone who is perceived as interested and concerned may directly ease some of the affective aspects of worry and anxiety such as fear-beliefs and catastrophisation surrounding their pain [66]. Patients who find a clinicians explanation of their problem credible and who find the proposed treatment plan believable are seen to have lower FAB and generally achieved better outcomes than those who do not [67,68]. In the group of chiropractic clinics involved in this study it is usual to include advice on coping with and managing their pain. For the majority of LBP patients presenting with mechanical back pain this advice would be expected to include key messages suggested by guidelines including; 'back pain whilst very painful is not caused by anything medically serious', 'activities that increase back pain are unlikely to be doing more damage', and 'the quicker you return to normal activities the faster you will get better' [69]. These and similar messages have been developed specifically to address anxiety, fear-avoidance beliefs and catastrophic thinking in patients regarding their back pain.

When reviewing the role of reassurance in the management of patients in pain Linton et al concludes that reassurance is a complex process involving an interaction of patient experience, thoughts and beliefs, and emotions [70]. Further, they report that it has a more positive and lasting effect on patients who present with lower levels of worry, a group that the limited evidence to-date suggests, may include those patients presenting to chiropractors.

Clear limitations exist in this study. One is the fact only a restricted population from a group of linked clinics were investigated. Sampling bias and clustering effects strongly limit the generalisability of these results. In addition the absence of a control group precludes any causative relationships between improvement in symptoms and treatment. Further prospective matched studies are called for, with larger patient samples from a wider cohort of practitioners to investigate possible components of consultation that may modify psychological variables, reassure patients or reduce non-physical barriers to recovery.

## Conclusions

In this study higher **pre-visit **catastrophisation was moderately associated with poor short-term outcome in patients presenting to chiropractors with lower back pain. In contrast, **post-visit **catastrophisation, pain related self-efficacy, fear-avoidance beliefs and negative back beliefs had a significant influence on outcomes.

## Competing interests

The authors declare that they have no competing interests.

## Authors' contributions

JF conceived of the study, and was involved with its design, data collection, statistical analysis, interpretation and drafting the manuscript. DN performed the statistical analysis and was involved with its interpretation and in drafting the manuscript. PM participated in designing the study, interpreting the data and in drafting the manuscript. All authors read and approved the final manuscript.
